# Lipid metabolism does not influence the expression of proximal aortopathy in bicuspid aortic valve disease

**DOI:** 10.1186/1749-8090-10-S1-A65

**Published:** 2015-12-16

**Authors:** Tatiana M Sequeira Gross, Thomas Kuntze, Evaldas Girdauskas

**Affiliations:** 1 Department of Cardiac Surgery, Central Clinic Bad Berka, Germany

## Background/Introduction

Controversy exists regarding the pathogenesis of bicuspid aortic valve (BAV) associated aortopathy. Recent data indicate the potential role of lipid metabolism in the expression of aortopathy.

## Aims/Objectives

We aimed to correlate the markers of lipid metabolism with severity of proximal aortopathy in patients with BAV vs. tricuspid aortic valve (TAV) disease.

## Method

A total of 458 consecutive patients (mean age 64 ± 11 years, 68% male) underwent aortic valve replacement (AVR) with/without proximal aortic surgery from January,2008 through December,2014. All patients undergoing combined procedures (e.g., AVR+CABG) were excluded. Only patients in whom proximal aortic dimensions were defined by preoperative CT/MRI and/or TOE were included. Correlation analysis was performed between markers of lipid metabolism (i.e., cholesterol, LDL, HDL, and triglyceride) and maximal diameter of the proximal aorta in BAV subgroup (n = 273) vs. TAV subgroup (n = 185). Moreover, we compared correlation patterns between markers of lipid metabolism and maximal aortic diameter in BAV insufficiency (n = 46) vs. BAV stenosis (n = 227) cohorts. Logistic regression was performed to identify risk factors for proximal aortic diameter >40 mm in BAV and TAV subgroups.

## Results

No correlation was found between markers of lipid metabolism and proximal aortic diameter in BAV subgroup (r = -0.1, p = 0.1) and TAV subgroup (r = 0.006, p = 0.9). No significant differences in correlation patterns were found between markers of lipid metabolism and maximal aortic diameter in BAV insufficiency (r = 0.03, p = 0.8) vs. BAV stenosis (r = -0.1, p = 0.1) cohorts. Logistic regression analysis revealed triglyceride levels (HR 1.4, p = 0.05) and statin therapy (HR 0.4, p = 0.03) as predictors of proximal aortic diameter >40 mm in TAV subgroup only.

## Discussion/Conclusion

Our study demonstrates no linear correlation between markers of lipid metabolism and proximal aortic diameters in a surgical cohort of BAV and TAV patients. Statin therapy and triglyceride levels influence significantly proximal aortic diameter in patients with TAV, but not with BAV disease.

